# Digital holographic high-speed 3D imaging for the vibrometry of fast-occurring phenomena

**DOI:** 10.1038/s41598-017-10919-5

**Published:** 2017-09-05

**Authors:** Takashi Kakue, Yutaka Endo, Takashi Nishitsuji, Tomoyoshi Shimobaba, Nobuyuki Masuda, Tomoyoshi Ito

**Affiliations:** 1 Graduate School of Engineering, Chiba University, 1-33 Yayoi-cho, Inage-ku, Chiba, 263-8522 Japan; 2 Institute of Science and Engineering, Kanazawa University, Kakuma, Kanazawa, 920-1192 Japan; 3 Department of Applied Electronics, Tokyo University of Science, 6-3-1 Niijuku, Katsushika-ku, Tokyo, 125-8585 Japan

## Abstract

Digital holography allows production of high-speed three-dimensional images at rates over 100,000 frames per second; however, simultaneously obtaining suitable performance and levels of accuracy using digital holography is difficult. This problem prevents high-speed three-dimensional imaging from being used for vibrometry. In this paper, we propose and test a digital holography method that can produce vibration measurements. The method is based on single-shot phase-shifting interferometry. Herein, we imaged the surface of a loudspeaker diaphragm and measured its displacement due to the vibrations produced by a frequency sweep signal. We then analyzed the frequency of the experimental data and confirmed that the frequency spectra inferred from the reconstructed images agreed well with the spectra produced by the sound recorded by a microphone. This method can be used for measuring vibrations with three-dimensional imaging for loudspeakers, microelectromechanical systems, surface acoustic wave filters, and biological tissues and organs.

## Introduction

High-speed and non-invasive imaging techniques are important for elucidating the size, shape, motion and function of biological tissues and organs^1–10^. Vibration measurement, or vibrometry, techniques are useful for evaluating the health of the hearing organs of most vertebrates^3, 4^ and elucidating the high-efficiency wing-beat mechanisms of insects^7^. Stimulated Raman scattering (SRS) microscopy^1, 5^ and optical coherence tomography (OCT)^2, 4, 10^ have been researched as potential biomedical imaging techniques. However, achieving the high-speed three-dimensional (3D) imaging at the rate of more than 100,000 frames per second is difficult with these techniques because SRS microscopy requires long exposure times to obtain signal information as the signals are quite weak. Contrarily, OCT requires a scanning process to achieve 3D images. Herein, we focus on holography^11–14^. Holography can record both in-plane and depth 3D information as a hologram, an interference fringe pattern, using the interference of light with a single-shot exposure. In a reconstruction process, the 3D information of a recorded object can be reconstructed by adequately illuminating a hologram by using diffraction of light; in other words, holography can record and reconstruct the wavefronts of light, which include not only the light intensity but also the phase distributions.

Although it is difficult for classical holography, which needs a holographic recording material to record a hologram, to reconstruct 3D motion pictures of fast phenomena, digital holography (DH)^15–38^ can overcome the problem; this is because DH records holograms with an image sensor capable of capturing images in the form of a video, such as a charge-coupled device or complementary metal–oxide–semiconductor, instead of a holographic recording material that requires more time to rewrite itself as compared to an image sensor or can be exposed only once. This enables DH to measure the 3D motion of the fast phenomena that occur in cells and organs^20, 28, 36^, living organisms^24, 29^, particles^17^, gas flow^27^, shock waves^21^, sound waves^34, 37^ and discharges^30^.

Herein, we aim to use DH to simultaneously perform high-speed 3D imaging and vibrometry. Laser Doppler vibrometry^26^ is a common technique for vibrometry, and it can perform at high speeds; however, laser Doppler vibrometers record interference patterns using a single-pixel detector, so these devices cannot produce 3D images. Conversely, digital holographic vibrometry^16, 18, 19, 23, 36^ can realize 3D imaging.

Generally, vibration displacements are smaller at high frequencies than at low frequencies; as a result, DH systems need to be very accurate in the direction of the depth of the DH to be used for high-speed 3D vibrometry. The accuracy of measurement of conventional DH systems, however, is insufficient for precise 3D measurement of fast phenomena due to of the superposition of noise terms like 0^th^ order diffraction waves and conjugate images. Yu *et al*. designed a 3D DH system that successfully measures vibration using Fourier transforms and spatial filtering to remove noise terms^18, 19^. Their Fourier filtering method can detect the desired term from a recorded hologram because the desired term can be easily separated from the noise terms in Fourier space when the hologram resolution is sufficiently high. The resolution of an image captured by an image sensor, however, generally decreases with increasing frame rates. Therefore, production of accurate high-speed 3D vibrometry using Fourier filtering is difficult because of the low resolution of digital holograms. We, therefore, used a single-shot phase-shifting DH technique based on space-division multiplexing, known as parallel phase-shifting DH^39^. This technique is capable of producing 3D measurements that are more accurate than those produced using Fourier filtering^40^. Although several studies have reported successful high-speed 3D imaging of dynamic phenomena using parallel phase-shifting DH^25, 27, 29, 30, 33, 35, 37, 38^, no study has reported simultaneous high-speed 3D imaging and vibrometry by parallel phase-shifting digital holography for vibration measurements, to the best of our knowledge. Ney *et al*. developed a digital holographic vibrometry technique that could operate at 500 kHz^38^; however, they only used single-pixel detectors to record the interference patterns and did not achieve 3D imaging. We report the first experimental confirmation that high-speed 3D imaging can be used for vibrometry by parallel phase-shifting DH for the first time.

## Results

The greatest advantage of our high-speed 3D imaging vibrometry technique is that it can measure the vibration displacement of not only stationary vibrations but also of non-stationary vibrations. In order to demonstrate this advantage, we recorded holograms by inputting a frequency-swept signal into a loudspeaker (which was the object in our experimental setup). We generated the frequency-swept signal as a waveform audio format (WAV) file, and its frequencies were swept linearly from 0 to 20 kHz in 1.2 s. By playing the WAV file on a host computer, we input the frequency-swept signal from the computer into the loudspeaker *via* the computer’s headphone jack. The maximum amplitude of the signal was adjusted so as to be ~200 mV.

**Figure 1:**
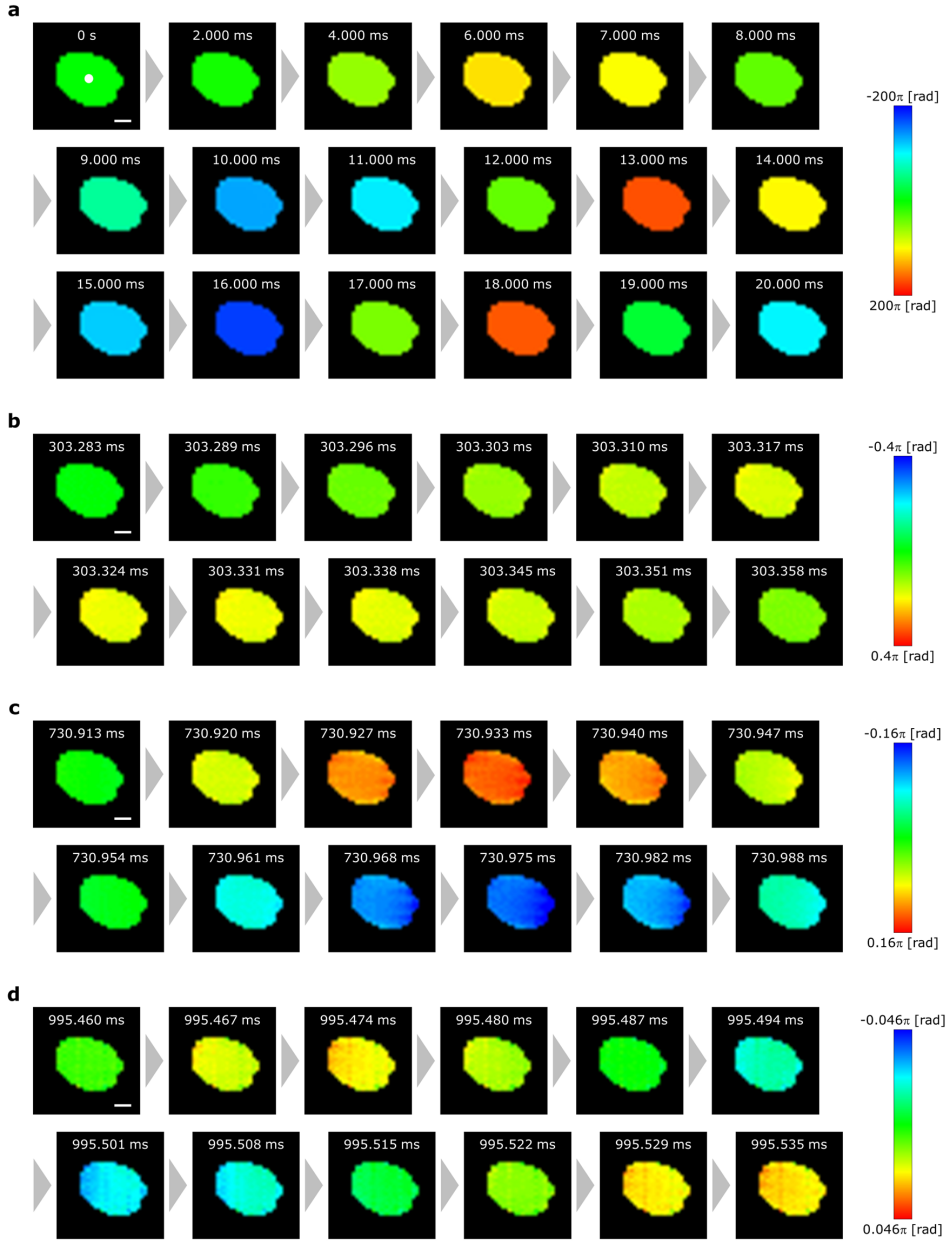
Reconstructed phase distributions. (**a**) Images from *t* = 0–20 ms. (**b**) Images from *t* = 303.283–303.358 ms. (**c**) Images from *t* = 730.913–730.988 ms. (**d**) Images from *t* = 995.460–995.535 ms. The scale bars in the figure are 100 μm long.

**Figure 2:**
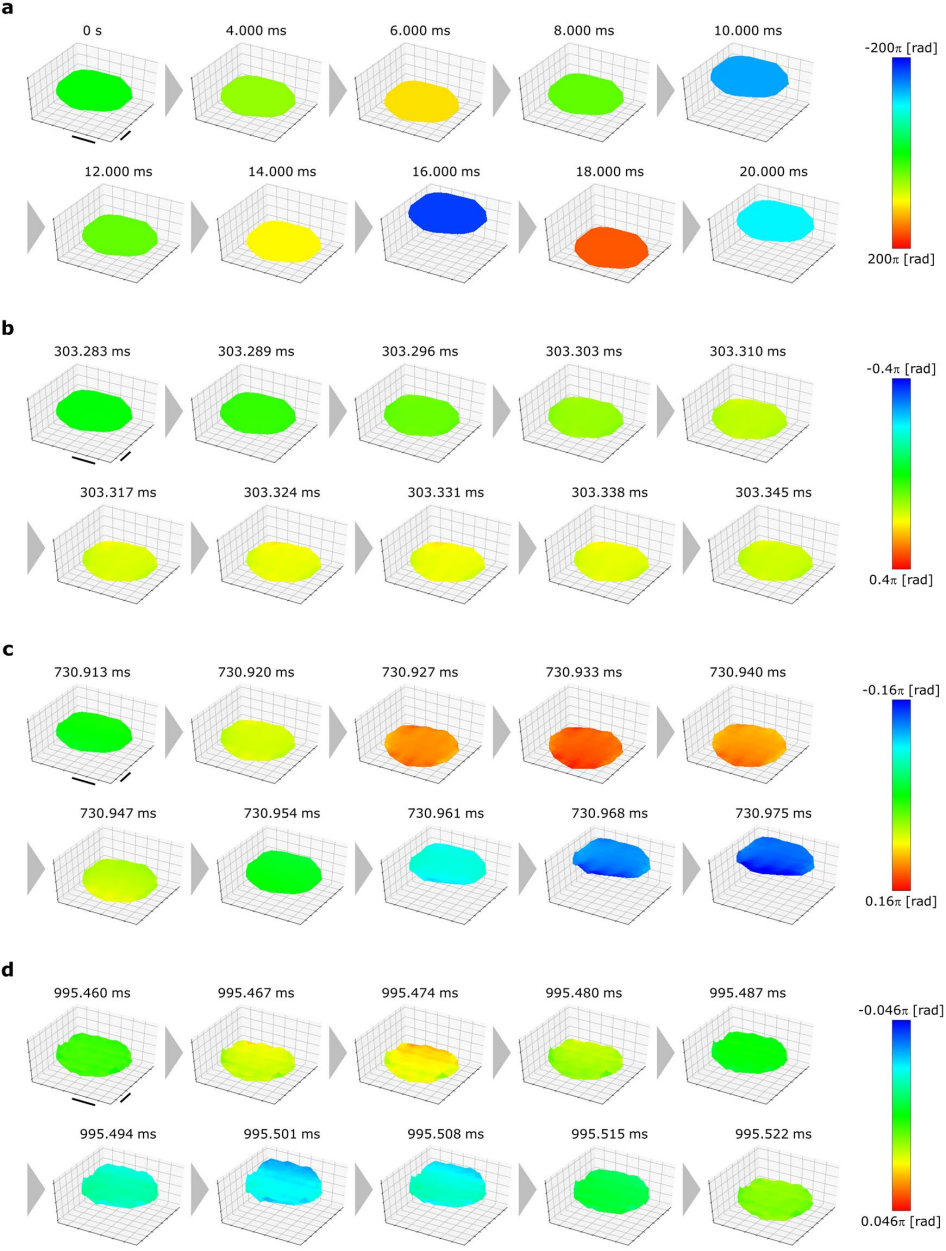
3D profiles of the reconstructed phase distributions. (**a**) Images for *t* = 0–20 ms (Supplementary Video S1). (**b**) Images for *t* = 303.283–303.345 ms (Supplementary Video S2). (**c**) Images for *t* = 730.913–730.975 ms (Supplementary Video S3). (**d**) Images for *t* = 995.460–995.522 ms (Supplementary Video S4). The scale bars on horizontal axes in the figure are 100 μm long.

Figure 1 shows parts of the reconstructed motion images that were focused on the surface of the loudspeaker diaphragm when the frequency-swept signal was input into the loudspeaker. We defined the time when the vibration started as being *t* = 0 s. The images in Fig. 1 indicate the phase distributions of the object wave; half of the phase values corresponded to the vibration displacement of the diaphragm. Because it is difficult to obtain the correct phase distribution in situations where the intensity of the object wave is weak, we filtered the phase distributions by using the binarized intensity distribution and set the colour of the low-intensity pixels to black. Additionally, in each figure, we subtracted the phase values of the reconstructed images before the signal input from those of each of the reconstructed images after the signal input so as to set the phase values of the static states to zero. As the reconstructed phase values are wrapped onto the range −π to π in DH, we could not obtain correct phase values without a phase-unwrapping method. We thus applied a temporal phase-unwrapping method^41^ to the reconstructed phase distributions after the subtractions in Fig. 1. Figure 2 and Supplementary Movies show the 3D profiles of Fig. 1. The time variation of the vibration displacement of the loudspeaker diaphragm can clearly be observed, and the image does not contain speckle noise. We were also able to observe that the frequency of the vibration displacement increased over time. To the best of our knowledge, the achieved frame rate is the fastest for single-shot phase-shifting DH for high-speed 3D imaging vibrometry.

**Figure 3:**
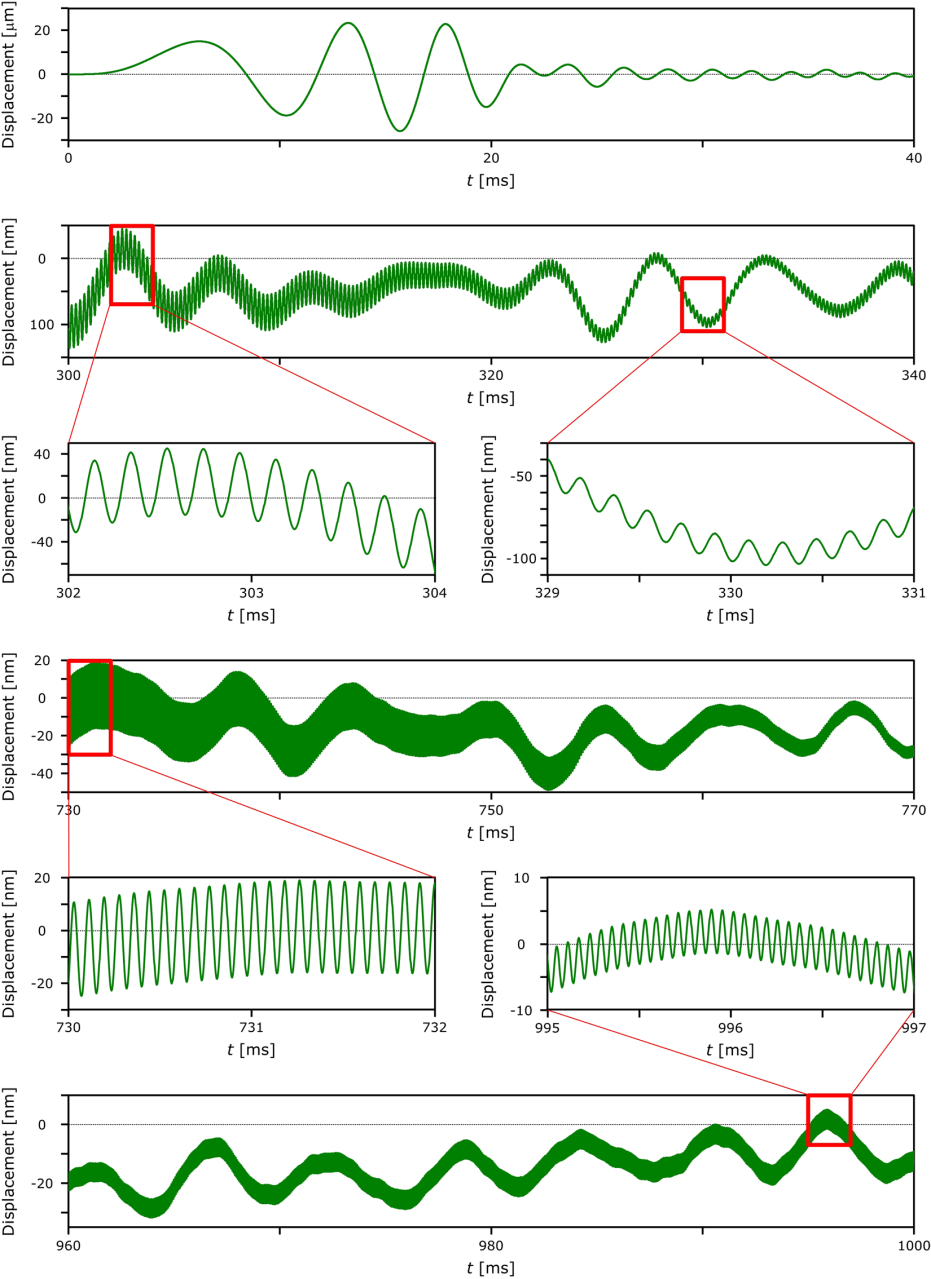
Parts of the time variation of the vibration displacement at a certain pixel on the reconstructed images.

For the analysis of the experimental results, we focused on the time variation of the displacement. Figure 3 shows parts of the time variation of the vibration displacement at the single pixel marked by a white dot in Fig. 1a. We observed that there was a large displacement associated with the low-frequency values until *t* = ~10 ms. This was mainly caused by the initial motion of the diaphragm. It is difficult for conventional digital holographic vibrometry to detect and measure transient vibration that is slow but has a large displacement. This is because phase values are wrapped, and correct displacements are not measured when the vibration displacement between neighboring frames is larger than 2π; therefore, higher frame rates are required for decreasing the displacement between neighboring frames, even when the vibration frequency is low. Our system can measure the large displacement caused by initial motion with transient vibration using high-speed 3D imaging. Figure 3 shows that the smallest amplitude of the vibration displacement was ~3.2 nm; this means that our system was able to detect and measure displacements that had amplitudes of at least 3.2 nm; we also found that the vibration displacement contained some periodicity, and the frequency of the displacement increased over time.

**Figure 4:**
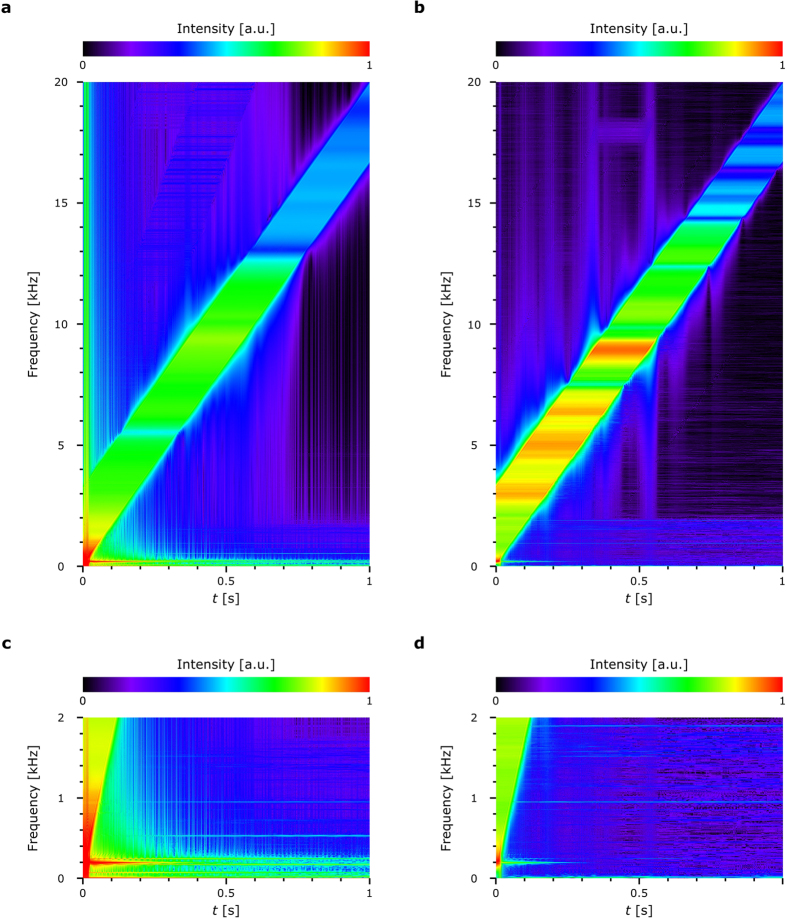
Results of the frequency analysis. (**a**) Spectra of the reconstructed images. (**b**) Spectra of the recorded sound. (**c**) and (**d**) Are the enlarged views of (**a**) and (**b**), respectively.

We then performed a frequency analysis by applying a fast Fourier transform (FFT) to the vibration displacement that was obtained. Figure 4a shows the result of the frequency analysis from *t* = 0–1 s. We applied each FFT to 116,250 frames per *t*; in the case of *t* = 500 ms, for example, we used 116,250 frames between *t* = 500–700 ms for the FFT. We normalized the intensity values between 0 and 1 after taking their common logarithm to easily recognize the frequency spectra. It can be seen that the high-intensity range shifted linearly from 0 Hz to 20 kHz over time. As the frequency was swept from 0 Hz to 20 kHz over 1.2 s, and because 116,250 frames were used per FFT, the width of the high-intensity range was estimated as being ~3.3 kHz. In Fig. 4a, the width of the range can be also estimated as being ~3.3 kHz. We found that the intensity values at the high frequency values were lower than those at the low frequencies in Fig. 4a; this is because the displacement at high frequencies generally decreased more than at low frequencies.

Figure 4b shows the results of the frequency analysis performed on the sound recorded by the microphone. We can see that the trend in Fig. 4a agrees well with that observed in Fig. 4b. We therefore believe that we successfully demonstrated that our technique was able to realize high-speed 3D imaging that could be used for the vibrometry of fast phenomena. In addition, while we demonstrated that our system can image and measure the vibration displacement of a loudspeaker diaphragm, we also believe that it can estimate the frequency spectra of the sound output by the loudspeaker.

## Discussion

Figure 3 also shows that the reconstructed motion images had some low-frequency vibration displacements that may not have been caused by the sweep signal. Figure 4c and d are the enlarged views of Fig. 4a and b, respectively, in the range of 0–2 kHz. Figure 4c shows that there were some strong peaks at low frequencies, such as at ~200 Hz, ~530 Hz and ~955 Hz, throughout the inputting of the frequency-swept signal. We estimate that the vibration at ~200 Hz was caused by the initial motion of the diaphragm, because the intensity gradually decreased over time; furthermore, Fig. 4d also has a strong peak at ~200 Hz. The strong peak at ~530 Hz does not appear in Fig. 4d, and the intensity of this peak does not change over time; we, therefore, believe that this peak was caused by some unknown disturbance in the experimental environment. The strong peak at ~955 Hz also appeared in Fig. 4d; however, the intensity of this peak did not change during the inputting of the frequency-swept signal. We, therefore, checked the sound caused by the rotation of the cooling fan of the high-speed polarization-imaging camera and determined that this peak was due to a vibration and sound caused by the rotation. Additionally, Fig. 4d has a peak at ~1.95 kHz, which cannot be seen in Fig. 4c; this implies that this peak was due to the second-harmonic sound of ~955 Hz.

There are some differences between the frequency spectra due to the frequency-swept signals in Fig. 4a and b. The most notable discrepancy is at ~5.5 kHz, where Fig. 4a has a local minimum but Fig. 4b does not. As we did not accurately calibrate some of the devices used in the experimental setup (*i.e*. the loudspeaker, the microphone and the sound card on the host computer) in the experiment, these discrepancies may have been not caused by parallel phase-shifting DH but by these devices. However, we nevertheless believe that our experimental results are noteworthy, because they indicate that our system enables the measurement of vibration displacements that cannot be measured by sound recorded by a microphone. In the future, we will aim to demonstrate this specific ability, as not only is it useful for analysing and evaluating the performance of a loudspeaker, but it could also be useful for other vibration media, such as microelectromechanical systems, surface acoustic wave filters and biological tissues and organs.

Although we successfully demonstrated that our technique can capture high-speed 3D images that can be used for vibrometry, we found that the measurable area of the loudspeaker diaphragm in this experiment was too narrow to image as well as to measure the mode profiles of the vibrations. We believe that this was due to the power of the optical source used in this experiment being insufficient. We believe that this problem will be solved through the use of an optical source that has a greater intensity; furthermore, a demagnification of the optical system would be able to produce a wider measurable area.

## Methods

### Theory

Phase-shifting DH^42^ enables highly accurate measurements to be made, because of to its use of phase-shifting interferometry. In this type of interferometry, multiple (generally more than two) holograms are recorded by the phase of a reference wave being shifted sequentially. For the sake of simplicity, suppose that the number of phase-shift steps is four, and the phase-shift value is π/2. Four holograms at (*x*, *y*, 0), which indicate the coordinates of an arbitrary pixel on the image sensor plane, are denoted as *H*(*x*, *y*, 0; 0), *H*(*x*, *y*, 0; *π*/2), *H*(*x*, *y*, 0; *π*) and *H*(*x*, *y*, 0; 3*π*/2); they are recorded by phase-shifting DH. In our example, 0, π/2, π and 3π/2 indicate the values of the phase shifts of each reference wave. In the reconstruction process by the phase-shifting DH, *U*_0_(*x*, *y*, 0), which is the complex amplitude distribution of the object wave at (*x*, *y*, 0), is calculated using the four recorded holograms. *U*_0_(*x*, *y*, 0) is given by the following equations:

1 $$\mathrm{Re}[{U}_{0}(x,\,y,\,0)]=\frac{H(x,\,y,\,0;\,0)-H(x,\,y,\,0;\,\pi )}{4r},$$

2 $$\text{Im}[{U}_{0}(x,\,y,\,0)]=\frac{H(x,\,y,\,0;\,3\pi /2)-H(x,\,y,\,0;\,\pi /2)}{4r}.$$

Here, $\mathrm{Re}[C]$ and $\text{Im}[C]$ indicate the real and imaginary parts of a complex number *C*, respectively, and *r* indicates the amplitude of the reference wave. By assuming that the reference wave is a plane wave that is introduced perpendicularly into the image sensor plane, *r* becomes constant on the image sensor plane; this results in the denominators of Eqs (1) and (2) becoming constant. Therefore, *U*_0_(*x*, *y*, 0) can be calculated by *H*(*x*, *y*, 0; 0), *H*(*x*, *y*, 0; *π*/2), *H*(*x*, *y*, 0; *π*) and *H*(*x*, *y*, 0; 3*π*/2). By applying a diffraction calculation^43^ to *U*_0_(*x*, *y*, 0), *U*_0_(*x*, *y*, *z*), which is the complex amplitude of the object wave at an arbitrary distance, can also be calculated.

Measuring dynamic phenomena is quite difficult for phase-shifting DH, because multiple phase-shifted holograms need to be recorded sequentially in order for phase-shifting interferometry to be applied. In order to overcome this problem, we proposed a method that used single-shot phase-shifting DH based on space-division multiplexing (*i.e*. parallel phase-shifting DH)^39^.

**Figure 5:**
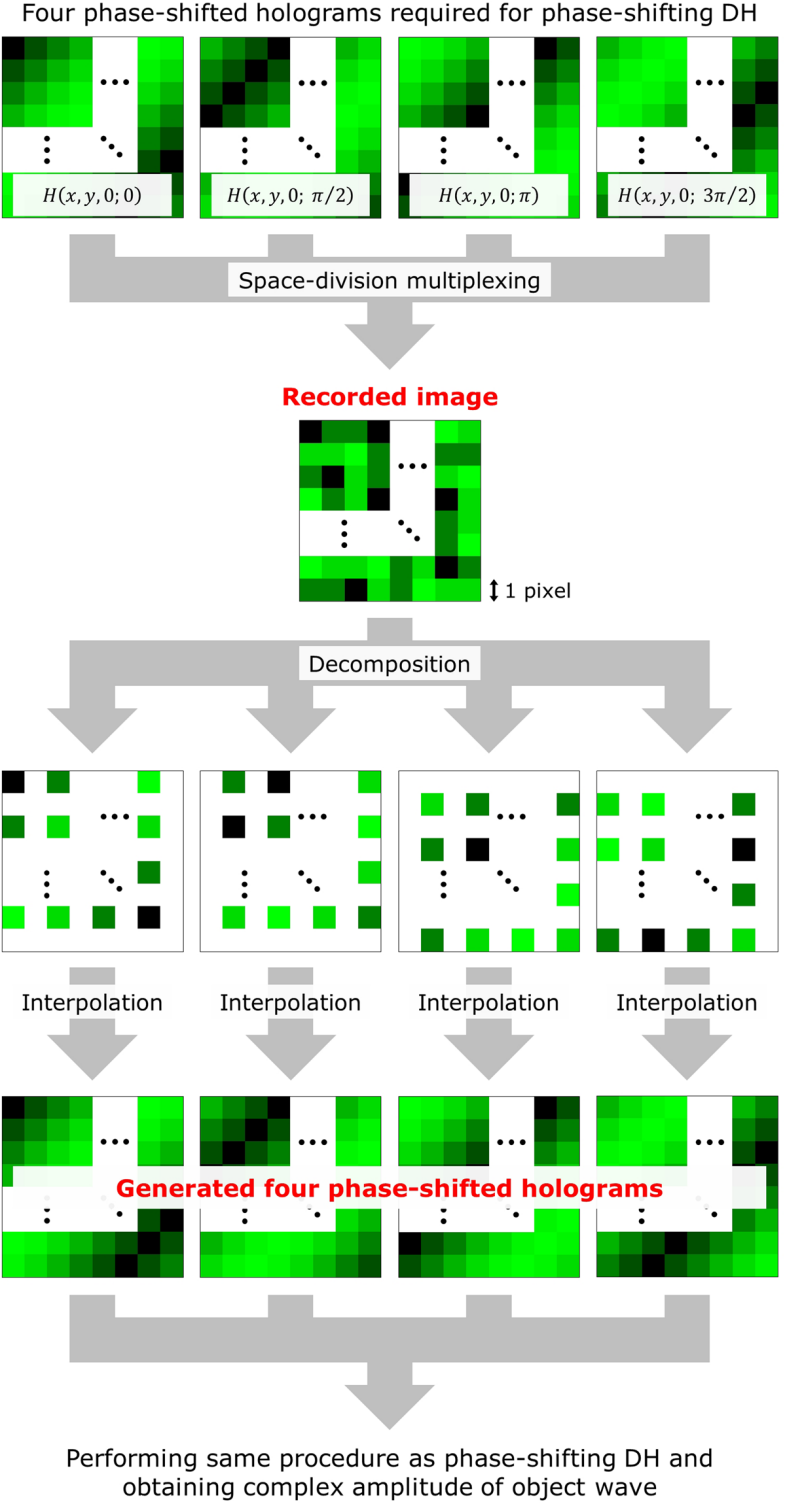
Flow of the reconstruction process in parallel phase-shifting digital holography when four phase-shift steps are used.

Figure 5 shows the flow of the reconstruction process of a parallel phase-shifting DH that utilizes four phase-shift steps. We recorded a single image, which included the data of the four phase-shifted holograms pixel by pixel. The pixels of the recorded image were decomposed into four different images with respect to the phase-shift values. Because the four decomposed images have vacant pixels, the pixel values of the vacant pixels are interpolated by the adjacent pixels. Four phase-shifted holograms are then generated by the interpolation process. After that, the same procedure as that used for the phase-shifting DH was applied to the generated holograms, which allowed for the complex amplitude of the object wave to be calculated.

In order to image the complex amplitude of the object wave, we needed to quantize the complex amplitude as a real number. The intensity and/or phase distributions of the complex amplitude are used in general because the intensity distribution corresponds to the image captured via photography or the human eye and the phase distribution has minute depth information. By using Eqs (1) and (2), we can calculate the intensity and phase distributions, *I*(*x*, *y*, *z*) and *P*(*x*, *y*, *z*), respectively, using the following equations:

3 $$I(x,\,y,\,z)={(\mathrm{Re}[{U}_{0}(x,y,z)])}^{2}+{(\text{Im}[{U}_{0}(x,y,z)])}^{2},$$

4 $$P(x,\,y,\,z)={\tan }^{-1}(\frac{\text{Im}[{U}_{0}(x,\,y,\,z)]}{\mathrm{Re}[{U}_{0}(x,\,y,\,z)]}).$$

## Experimental Setup

**Figure 6:**
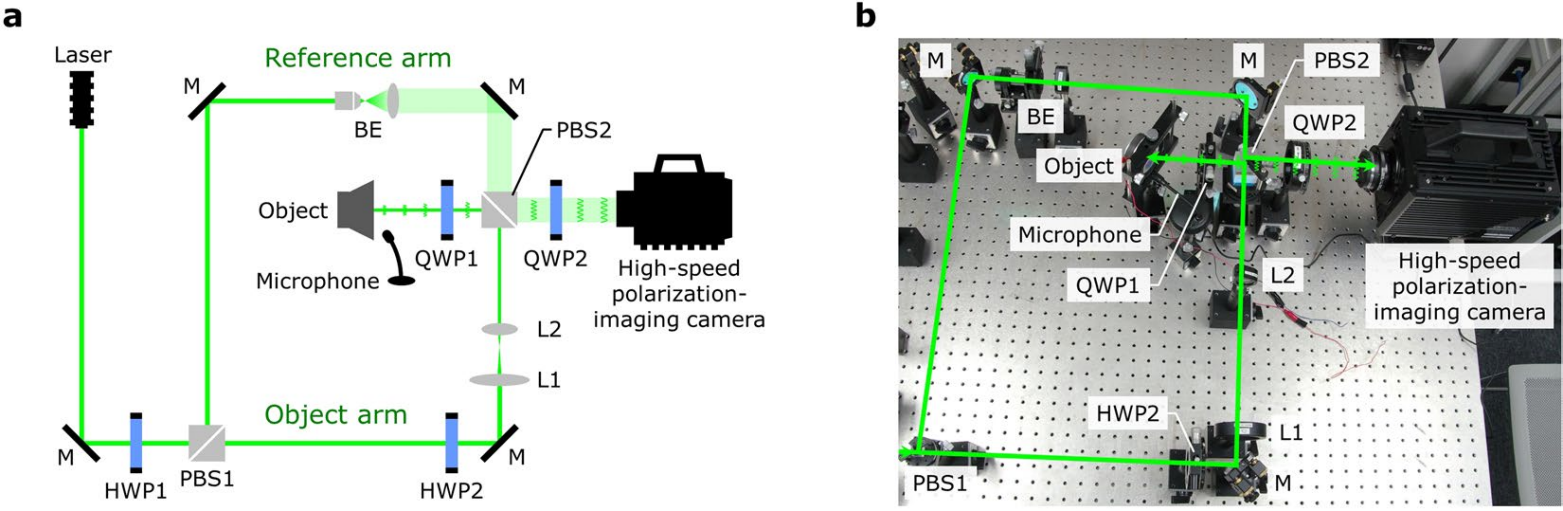
Experimental setup for the high-speed 3D imaging system used for vibrometry. (**a**) Schematic of the experimental setup. (**b**) Photograph of the experimental setup. In the figure, M represents mirrors, HWP represents half-wave plates, PBS represents polarization-beam splitters, L1 is the 150-mm focal length convex lens, L2 is the 50-mm focal length convex lens, QWP represents the quarter-wave plates and BE is the beam expander.

Figure 6a and b show the schematic and a photograph of the experimental setup, respectively. A 532-nm wavelength green laser (Showa Optronics, ‘J150GS’) was used as the optical source. The linearly polarized wave emitted from the laser was divided into two waves by the first polarization-beam splitter (PBS1); the ratio between the two waves was adjusted by the first half-wave plate (HWP1). The wave passing through the PBS1 is called an illumination wave, and it is introduced into the object arm; the wave reflected by the PBS1, meanwhile, is called a reference wave, and it is introduced into the reference arm.

In the object arm, the illumination wave passes through the second half-wave plate (HWP2); because the HWP2 rotates the polarization direction of the illumination wave by 90°, it results in the illumination wave being reflected by the second polarization-beam splitter (PBS2). We inserted a beam narrower, which consisted of two convex lenses (L1 and L2), between HWP2 and PBS2 in order to increase the illumination wave’s light intensity per unit area. The focal lengths of L1 and L2 were 150 and 50 mm, respectively. The illumination wave reflected by the PBS2 passes through the first quarter-wave plate (QWP1), and the fast (or slow) axis of the QWP1 was inclined so as to be 45° relative to the polarization direction of the original illumination wave. This resulted in the linear polarization of the illumination wave being converted into a circular polarization.

The illumination wave is then introduced into the object; in this study, we used a loudspeaker (Koizumi Musen, ‘70FB02BC’) as the object. In order to record the vibration displacement of the surface of the loudspeaker diaphragm, we set the wave that was reflected by the surface of the loudspeaker diaphragm as the object wave. Because the object wave passes through the QWP1, the circular polarization of the object wave is converted into a linear polarization; this results in the polarization direction of the object wave being orthogonal to that of the illumination wave. The object wave then passes through the PBS2. In the reference arm, the reference wave is introduced into the beam expander, which expands the beam diameter of the reference wave. The expanded reference wave is then reflected by the PBS2.

The object wave and the reference wave are then introduced into the second quarter-wave plate (QWP2). Because the fast (or slow) axis of the QWP2 is inclined to 45° relative to the polarization direction of the reference wave, both of the waves are converted into circular polarization waves. At this juncture, the rotating directions of the circular polarizations of the two waves are opposite, because the direction of the object wave is orthogonal to that of the reference wave.

Lastly, the object and reference waves are introduced into a high-speed polarization-imaging camera (Photron, ‘CRYSTA PI-1’). This camera has a polarization-detection function, which can select four polarization axes (0°, 45°, 90° and 135°) for a set of 2 × 2 pixels. It is able to record an interference fringe pattern that has four phase-shifted holograms pixel by pixel by using the polarization-detection function. The pixel pitch of the camera is 20 μm. We set the resolution (the number of pixels) of the holograms to 64 × 64 pixels. Because the camera can record 64 × 64 pixel images at a framerate of up to 581,250 frames per second, we set the frame rate to 581,250. The shutter speed was set to 1 μs, which corresponded to 1,000,000 frames per second. When the holograms were recorded by the camera, we also recorded the sound from the loudspeaker with a microphone (Audio-Technica, ‘AT9932PC’).

## Electronic supplementary material

Supplementary Movie S1

Supplementary Movie S2

Supplementary Movie S3

Supplementary Movie S4
